# Filling Polyoxoanions into MIL-101(Fe) for Adsorption of Organic Pollutants with Facile and Complete Visible Light Photocatalytic Decomposition

**DOI:** 10.3390/molecules27113404

**Published:** 2022-05-25

**Authors:** Qing Lan, Sujuan Jin, Bohan Yang, Zhiming Zhang, Xuyang Li, Haiquan Xie, Xiaoli Jin, Huan Zhang, Qiang Zhao

**Affiliations:** 1 Engineering Technology Research Center of Henan Province for Solar Catalysis, College of Chemistry and Pharmaceutical Engineering, Nanyang Normal University, Nanyang 473061, China; lanqnynu@163.com (Q.L.); jinsujuan2018@126.com (S.J.); xclq2009@163.com (B.Y.); eryang1024@163.com (X.L.); xljin_nnu@sina.com (X.J.); zhaoqiang0522@126.com (Q.Z.); 2 Institute of New Energy Materials & Low Carbon Technology, School of Materials Science & Engineering, Tianjin University of Technology, Tianjin 300384, China; zmzhang@email.tjut.edu.cn; 3 School of Science, Tianjin University of Science & Technology, Tianjin 300457, China

**Keywords:** polyoxometalates, metal–organic frameworks, composite materials, photocatalysis, antibiotics

## Abstract

Transition metal-substituted polyoxometalates (POMs) were filled into a metal–organic framework (MOF) to construct a series of POM@MOF composites (PMo_12_O_40_@MIL-101, PMo_11_VO_40_@MIL-101, PMo_10_V_2_O_40_@MIL-101). The composite materials possess ultra-high adsorption ability, especially for PMo_10_V_2_O_40_@MIL-101, with an adsorption capacity of 912.5 mg·g^−1^ for cationic antibiotic tetracycline in wastewater, much higher than that of isolated MIL-101(Fe) and the commonly used adsorption materials, such as activated carbon and graphene oxide. In particular, they can be used as efficient photocatalysts for the photodegradation of antibiotics under visible light irradiation. The complete photodegradation of the adsorbed species can induce the facile reusability of these composites for multiple cycles. This work opens an avenue to introduce POMs into an MOF matrix for the simultaneous adsorption and photodegradation of antibiotics.

## 1. Introduction

Currently, antibiotics are widely used in medicine, animal husbandry, and aquaculture due to their efficient bactericidal/bacteriostatic effects. However, the abuse of antibiotics and their residues has caused serious pathological damage and environmental effects, which have become one of the key issues to be urgently addressed [1,2,3,4,5,6]. Nowadays, more and more attention is paid to removing antibiotic residues, and various methods have been developed in this field, including membrane separation, biodegradation, photocatalysis, adsorption, and various oxidation methods [7,8,9,10,11,12,13,14]. Compared with other technologies, adsorption is considered to be one of the most excellent and facile methods to sequestrate antibiotic residue from water due to its low cost, easy operation, and high efficiency [15,16,17]. In this field, a series of materials have been explored as adsorbents for wastewater treatment, such as activated carbon, graphene oxide, and smectite [18,19,20]. It is evident from a literature survey that porous materials can function as useful adsorbents [21,22,23]. However, the existing adsorbents usually possess low adsorption capacity, a long cycle, and non-regeneration, which have become the key technical bottlenecks restricting the treatment of antibiotic pollution. Therefore, it is extremely desirable to develop facilely renewable adsorbents with ultra-high adsorption capacity for wastewater treatment, but this is still a very challenging task.

Polyoxometalates (POMs) are a group of polymetallic oxygen clusters, composed of transition metal ions (e.g., W^6+^, Mo^6+^ and V^5+^) and oxygen atoms. Their properties can be easily adjusted by choosing different metal elements and changing their structures, which makes them suitable for a wide range of applications in many fields, such as catalysis, photocatalysis, biological medicine, energy conversion, sensors, and magnetism [24,25,26,27,28,29,30,31,32,33,34,35]. Transition metal-substituted POMs, replacing one or more polyhedral {MO_x_} (M = W^6+^ and Mo^6+^) with other low-valence metal cations (e.g., V^5+^, Al^3+^, Cu^2+^ and so on), usually possess a high negative charge for the enrichment of cationic dyes and active sites for photocatalysts. However, the wide application of pure POMs has been largely hindered because of their inherent characteristics of small surface area (<10 m^2^_·_g^−1^) and high solubility in aqueous solution, which is inconvenient for the recovery and recyclability process [36,37]. Therefore, it is critical to explore a suitable solid matrix to immobilize POMs to greatly enhance the absorption ability of target organic contaminants with excellent recyclability [38].

Metal–organic frameworks (MOFs) are a particular class of crystalline materials, which are constructed by bridging metallic ions/clusters with organic linkers into periodic promising porous materials [39,40,41,42,43,44,45,46,47]. Embedding POMs in the porous MOF matrix can achieve heterogeneous adsorption and catalysis; in particular, the combination of two ideal molecules can afford unexpected performance. In this field, the composite materials formed by embedding POMs in the MOF matrix have been used for aerobic decontamination, solar-driven water splitting, and so on [48]. Among these MOFs, MIL-101(Fe), with a high surface area, attractive porosity, and stability in water, represents an ideal material for encapsulating POMs to construct composite absorbents [49,50,51].

**Figure 1:**
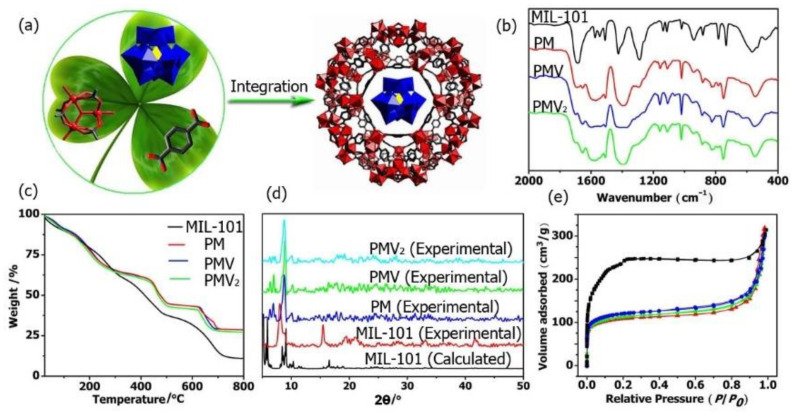
(**a**) One-pot synthesis of POM@MOF; (**b**) IR, (**c**) TG, (**d**) PXRD patterns of MIL-101, PM, PMV, and PMV_2_; (**e**) N_2_ adsorption patterns of MIL-101 (black ■); PM (red ▲); PMV (green ▼), and PMV_2_ (blue ●).

Herein, a series of POM@MOF composites, PMo_12_O_40_@MIL-101 (PM), PMo_11_VO_40_@MIL-101 (PMV), and PMo_10_V_2_O_40_@MIL-101 (PMV_2_), with superior adsorption ability for cationic antibiotics were designed and synthesized (Figure 1a). The adsorption capacity of PMV_2_ can reach as high as 912.5 mg·g^−1^, significantly superior to that of commonly used adsorption materials, such as activated carbon and graphene oxide. In particular, they can be used as efficient photocatalysts for the photodegradation of tetracycline (TC) under visible light irradiation. The complete degradation of adsorbed species induces the facile reusability of these composites for multiple cycles. The resulting PMV_2_ represents the first POM@MOF composite that is an efficient adsorbent with facile reusability via photoregeneration. This work opens an avenue to introduce POMs into the MOF matrix for the simultaneous adsorption and photodegradation of antibiotics.

## 2. Experimental Section

### 2.1. Synthesis of POM@MIL-101(Fe) Composites

The MIL-101(Fe) was synthesized according to the literature method [52] from FeCl_3_·6H_2_O, H_2_bdc and DMF by the solvothermal method at 383 K for 24 h. The experimental process of POM@MIL-101 was similar to that of MIL-101(Fe), except that 0.2 g POM (H_3_PMo_12_O_40_, H_4_PMo_11_VO_40_, H_5_PMo_10_V_2_O_40_) was added during the synthesis of MIL-101(Fe).

### 2.2. Antibiotic Adsorption Examinations

The antibiotic adsorption examinations were performed in a 100 mL flask. Prior to the adsorption examinations, the necessary activation of POM@MOF adsorbents was required by heating these composites in a vacuum at 323 K for 24 h. In the adsorption experiment, 2 mg of POM@MIL-101 absorbent was added to the antibiotic TC solution (10–40 mg·L^−1^, 50 mL) with stirring at room temperature, and the concentration change of the antibiotic was determined by the UV–Vis spectrum.

### 2.3. Calculation of Adsorption Capacity and Adsorption Rate (Adsorption%)

The adsorption capacity *Q*_eq_ (mg·g^−1^) and adsorption rate (adsorption%) were calculated according to the following equations:

$$Q_{\mathrm{eq}}=\frac{\left(C_{0}-C_{\mathrm{eq}}\right)V}{m}$$

$$\mathrm{Adsorption}\%=\frac{\left(C_{0}-C_{t}\right)\times 100\%}{C_{0}}=\frac{\left(A_{0}-A_{t}\right)\times 100\%}{A_{0}}$$

where *C*_0_, *C*_eq_, and *C*_t_ (mg·L^−1^) are the TC concentration of initial, equilibrium, and at time *t*. *Q*_eq_ (mg·L^−1^) is the adsorption capacity of TC, when it reaches adsorption equilibrium. *A*_0_ and *A*_t_ represent the absorbance of TC before and after adsorption. *V* (L) is the volume of TC solution and *m* (g) is the dosage of the adsorbent.

### 2.4. Photoregeneration of PWV_2_

The photocatalytic decomposition of antibiotics was carried out in an instrument equipped with a closed cooling-water system at room temperature. A 300 W Xe lamp with a 420 nm cut-off filter served as the visible light source by top-irradiation, with a distance of 3–5 cm between the liquid surface and the lamp, with continuous stirring during irradiation, and TC was chosen as the degradation target antibiotic. The initial concentration of tetracycline solution was approximately 10–40 mg·L^−1^. The regenerated catalyst was facilely separated by centrifugation for the next cycle.

## 3. Results and Discussion

### 3.1. Characterization of POM@MOFs

The FT-IR spectra of the monomers including H_3_PMo_12_O_40_, H_4_PMo_11_VO_40_, H_5_PMo_10_V_2_O_40_, and MIL-101(Fe) were shown in Figure 1b and Figures S1–S3. The characteristic bands in the range of 1067–1063 cm^−1^, 966–961 cm^−1^, 870–864 cm^−1^, and 787–781 cm^−1^ related to *ν*_as_(P-O_a_), *ν*_as_(Mo=O_t_), *ν*_as_(Mo-O_c_-Mo), and *ν*_as_(Mo-O_e_-Mo) (e, edge-sharing oxygen atoms; c, corner sharing oxygen atoms) were observed for all the POMs [53]. In these FT-IR spectra of PM, PMV, and PMV_2_ composites, the characteristic bands corresponding to MIL-101 MOF and the POMs were all observed, demonstrating the presence of both MIL-101 and POMs in these POM@MOF composites. In the thermogravimetric (TG) curve, the weight loss of PM, PMV, and PMV_2_ composites was significantly lower than that of MIL-101 (Figure 1c). The differences in weight loss reveal that the Keggin polyoxoanions were successfully located in the MIL-101 framework. The PXRD patterns of the as-synthesized MOF and the composites are presented in Figure 1d. The diffraction peaks of MIL-101 implied high crystallinity, and the PXRD patterns were consistent with that calculated from the crystal data, indicating the successful synthesis of MIL-101 MOF. The PXRD patterns of isolated MIL-101 and the composites were very similar, indicating that the crystal structure of MOF was retained in the composites. The BET surface area of MOF and the composite was studied at 77 K by the N_2_ adsorption–desorption isotherm. As shown in Figure 1e, the adsorption capacity of the composites decreased obviously compared to that of the isolated MOF. Their Langmuir surface areas were estimated as 401.5 m^2^·g^−1^ (PM), 418.6 m^2^·g^−1^ (PMV), 473.4 m^2^·g^−1^ (PMV_2_), and 896.6 m^2^·g^−1^ (MIL-101) by applying the Langmuir equations. These results further proved the incorporation of POM into the MOF matrix.

**Figure 2:**
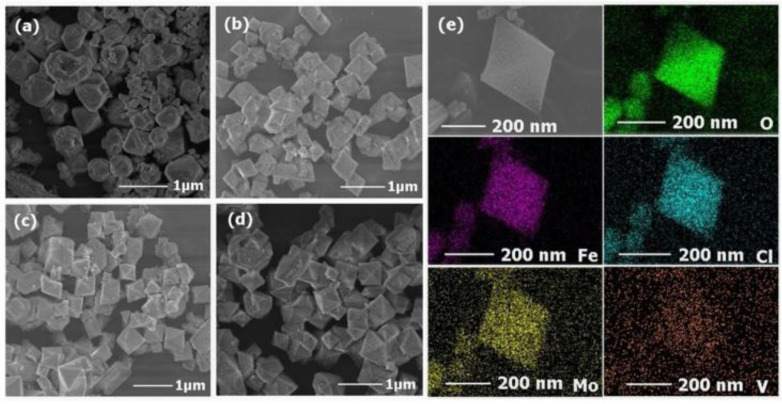
The SEM images of (**a**) MIL-101, (**b**) PM, (**c**) PMV, (**d**) PMV_2_ and (**e**) element mapping of PMV_2_.

The structure and morphology of the isolated MIL-101(Fe) nanoparticles and POM@MIL-101 composites were measured by field emission scanning electron microscopy (SEM). As shown in Figure 2a, MIL-101 nanoparticles were uniform polyhedral particles with diameters of 500–800 nm and a rough surface. After the encapsulation of POMs, the polyhedral morphology was well maintained in POM@MIL-101 and the average diameter decreased slightly (Figure 2). These results prove that the POM@MIL-101 maintained the framework structure of MIL-101. Further, energy-dispersive X-ray spectroscopy (EDS) elemental mapping images showed the uniform distribution of Mo, V, Fe, Cl, and O elements in these composites (Figure 2e and Figures S4–S6), revealing the encapsulation of POM in the MOF. All the above results indicate that the Keggin clusters were successfully encapsulated into the three-dimensional skeleton of the MIL-101 matrix, which maintained its structural integrity during the introduction of POMs.

### 3.2. Adsorption of Antibiotics

**Figure 3:**
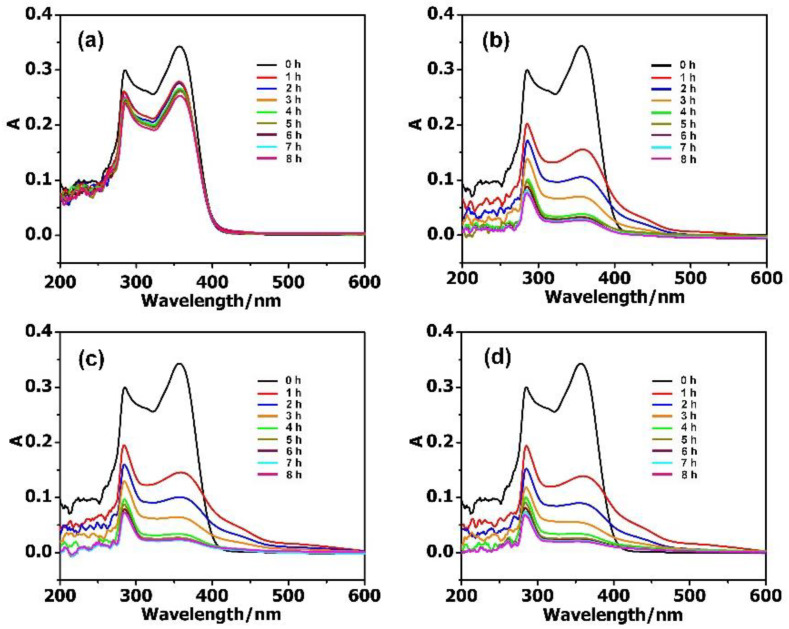
The UV–Vis spectra of 10 mg·L^−1^ TC during the adsorption stages using 2 mg (**a**) MIL-101; (**b**) PM; (**c**) PMV, and (**d**) PMV_2_.

The adsorption activities of MIL-101 and the composite materials towards antibiotics in aqueous solutions were systematically studied with UV–Vis absorption spectra. In the reaction vessel, 2 mg adsorbent was used for the removal of antibiotics, with the concentration ranging from 10 to 40 mg·L^−1^. As shown in Figure 3, the adsorption rate of antibiotic solution (10 mg·L^−1^, 50 mL) reached 88.35% for PM, 93.56% for PMV, and 95.34% for PMV_2_ in 8 h, while it was 27.03% for the isolated MIL-101. While increasing the concentration of antibiotics to 20 mg·L^−1^, the adsorption rate reached 88.70% for PM, 93.26% for PMV, 95.96% for PMV_2_, and a much lower adsorption rate of 20.34% for MIL-101 (Figure S7) in 8 h. These results illustrated that POM@MIL-101 had a much better absorption capacity than the isolated MIL-101.

**Figure 4:**
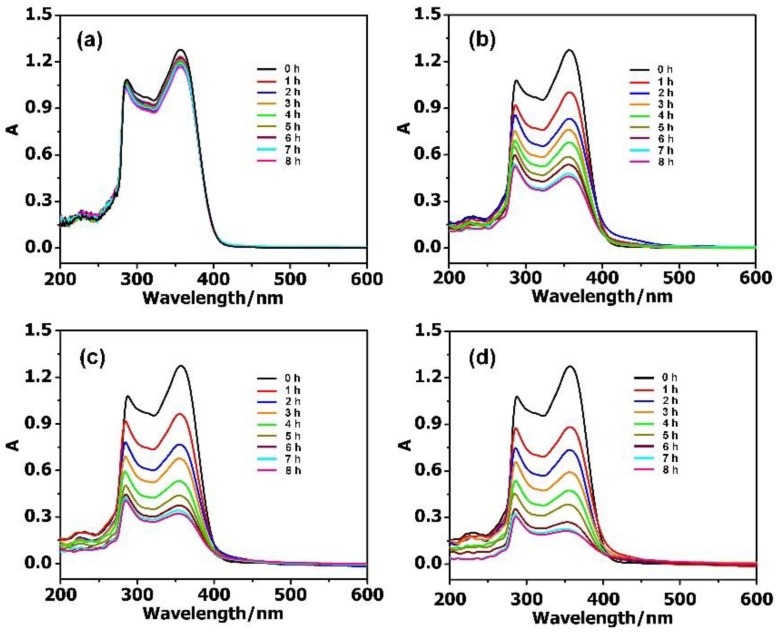
The UV–Vis spectra of 40 mg·L^−1^ TC during the adsorption stages using 2 mg (**a**) MIL-101; (**b**) PM; (**c**) PMV, and (**d**) PMV_2_.

By further increasing the concentration of the TC to 40 mg·L^−1^, the adsorption rate could reach 83.95% with 2 mg PMV_2_, 74.89% with PMV, and 64.76% with PM (Figure 4). Meanwhile, the adsorption rate for the isolated MIL-101 framework (≈9.16%) was much lower than that of the POM@MIL-101 composites. It can be clearly seen that the adsorption capacity of antibiotics increases when increasing their concentration and the negative charge of POMs. As shown in Table S1, when PMV_2_ (2 mg) was immersed in 40 mg·L^−1^ TC solution (50 mL), the absorption capacity reached 839.5 mg·g^−1^ within 8 h at room temperature, indicating its great advantages compared to activated carbon.

### 3.3. Effect of pH

**Figure 5:**
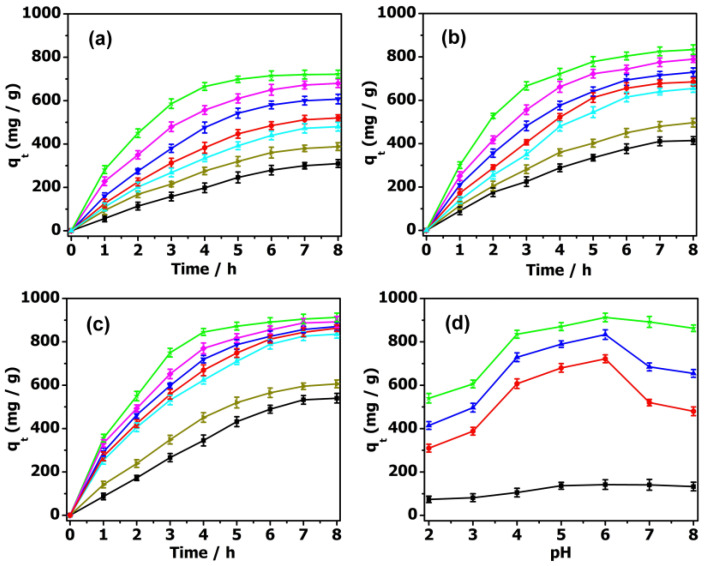
The effect of pH (pH = 2 black ■, 3 dark yellow ▶, 4 cyan ▲, 5 blue ▼, 6 green ★, 7 magenta ♦, 8 red ●) on TC adsorption by (**a**) PM, (**b**) PMV, (**c**) PMV_2_. (**d**) The effect of pH on TC adsorption by MIL-101 (black ■), PM (red ●), PMV (blue ▲), PMV_2_ (green ★).

In the design of an economical treatment system for wastewater, a high uptake capacity and fast adsorption rate are two important parameters for preparing highly efficient adsorbents. As is well known, the adsorption behaviors of these absorbents are highly dependent on the pH of the antibiotic solution. As shown in Figure 5, the pH of the TC aqueous solution was adjusted to 2–8 by dilute hydrochloric acid and sodium hydroxide solution to evaluate the pH influence on their absorption behavior. The adsorption capacity of PMV_2_ for TC was 540.0 mg·g^−1^ at pH = 2. With an increase in pH to 4, the adsorption capacity increased significantly to 835.2 mg·g^−1^. The maximum adsorption capacity of TC was shown at pH = 6, with a maximum adsorption capacity of 912.5 mg·g^−1^, and then gradually decreased as the pH rose to 8. When the pH value was lower than 3.3, it mainly existed in the form of a TCH_3_^+^ cation. Then, it mainly existed in the form of a neutral TCH_2_ molecule when the pH was between 3.3 and 7.7. As the pH was in the range of 7.7 to 9.8, it became TCH^−^ anions, and was converted into negative ions TC^2−^ after pH = 9.8 [23,54]. Since the surface charges of POM@MOF and TC are positive at pH = 2, electrostatic repulsion hinders the adsorption process, and the adsorption capacity is comparatively low. Meanwhile, the adsorption effect of POM@MOF is still higher than that of MOF, which is attributed to the possible hydrogen bonding between POMs and TC. In the process of increasing the pH to 4, TC mainly exists in the form of TCH_2_ and TCH^−^, and the adsorption capacity of the complex to TC increases sharply, and then remains almost constant until pH = 6. Since the POM in the acidic solution is protonated, it will produce a strong electrostatic attraction. It can be seen that the combination of electrostatic attraction, hydrogen bonding, and π–π interaction between POM@MOF and TC molecules leads to an increase in adsorption capacity. When the pH value is beyond 7, the electrostatic repulsion between TC^2−^ and the negative POM causes a decrease in adsorption capacity. According to the above discussion, we can conclude that the adsorption process of TC on POM@MOFs is the result of the interaction of electrostatic attraction, hydrogen bonding, and π–π interaction.

### 3.4. Evaluation of Visible Light Photocatalytic Performance

**Figure 6:**
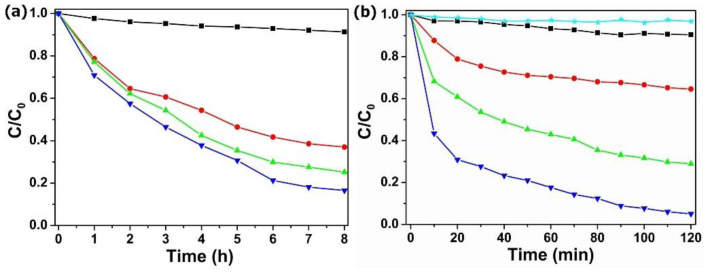
(**a**) The adsorption curves of TC solution by MIL-101 (black ■), PM (red ●), PMV (green ▲), and PMV_2_ (blue ▼) composites in the dark. (**b**) The removal of TC under visible light irradiation over no catalyst (cyan ★) MIL-101 (black ■), PM (red ●), PMV (green ▲), and PMV_2_ (blue ▼) composites.

Designing rapid wastewater disposal systems has significant economic value. Adsorption studies have found that POM@MOF have a good adsorption function, but the speed is slow, so the simultaneous adsorption and visible light photocatalytic process was determined in a relatively high concentration of TC solution (40 mg·L^−1^). As is shown in Figure 6a, MIL-101 showed very weak absorption in the TC solution. However, the POM@MOF composites exhibit remarkably enhanced TC adsorption properties, due to the introduced POM in the MOF frameworks. Figure 6b, shows the removal rate of TC under visible light irradiation over MIL-101 and POM@MOF. It is not difficult to see that the PMV_2_ showed stronger TC removal rates under visible light irradiation than the adsorption rates of TC solution in the dark in Figure 6a, which confirms the efficient visible light photocatalytic TC degradation of POM@MOF. The 10 mg·L^−1^ and 20 mg·L^−1^ TC photocatalytic degradation of PMV_2_ is shown in Figure S8. The results show that POM@MOF not only demonstrates a fast removal capacity and time saving, but it also can be reused.

The UV–Vis absorption spectra during the adsorption process and the PXRD were further observed to confirm the stability of the POM@MOF composites. It could be observed that no characteristic adsorption peaks of POM could be detected from the solution after the absorption (Figure S9), eliminating the possibility of the release of POMs from the MOF matrix. After the removal of TC, the PXRD of the POM@MOF composite was similar to that of the as-synthesized sample (Figure S10). All these results reveal the POM@MOF composite as a tool for the robust removal of antibiotics from water. The reusability of PMV_2_ for the removal of antibiotics was further studied through the recycle test. The photocatalyst was separated by centrifugation for reusability. As shown in Figure S11, when the PMV_2_ (2 mg) was added to the TC solution (50 mL 40 mg·L^−1^), the removal rate of the photocatalyst remained in line with that of the as-synthesized sample. These results demonstrate that POM@MOF composites not only have excellent removal properties for antibiotics, but also can be easily recycled via centrifugation for reusability.

**Figure 7:**
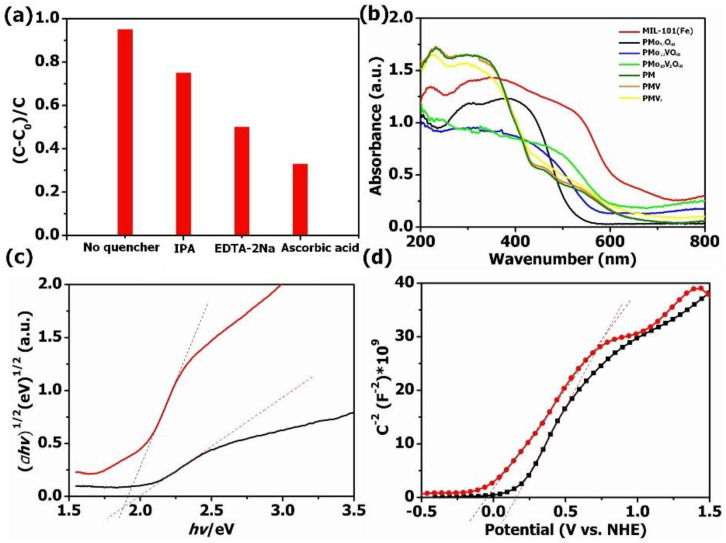
(**a**) The visible light degradation of TC applying PMV_2_ with different quenchers; (**b**) UV–Vis diffuse reflectance spectra; (**c**) the plots of (αhv)^1/2^ vs. (hv) for PMo_10_V_2_O_40_ and MIL-101(Fe); (**d**) the Mott–Schottky plots for PMo_10_V_2_O_40_ and MIL-101(Fe).

The mechanism of the photocatalytic degradation of TC was verified by a free radical capturing experiment. In this work, ascorbic acid and isopropanol were used as ·O_2_^−^ and ·OH radical scavengers, while EDTA-2Na was used as a *h*^+^ radical scavenger to verify the dominant reaction species during the degradation procedure, as shown in Figure 7a. PMV_2_ composites showed obviously decreased visible light photocatalytic activity for TC elimination when EDTA-2Na and ascorbic acid were added to the TC solution, indicating the photo-induced holes, where ·O_2_^−^ species play important roles in the visible light photocatalytic TC degradation. We could also find that only slightly decreased photocatalytic activity for TC elimination was observed when isopropanol (IPA) was added to the TC solution, indicating the generation of a small amount of ·OH under visible light irradiation.

UV–Vis DRS were employed to reveal the photoabsorption ability of the obtained materials (Figure 7b). Obviously, all these samples have strong absorption in the region of 200–800 nm. After the introduction of MIL-101(Fe), the absorption edges of POM@MOF composites were red-shifted compared with pure POMs, which leads to improved photocatalytic performance. The band gap energy (Eg) of bare PMo_10_V_2_O_40_ and MIL-101(Fe) could be calculated based on Tauc’s formula (*αhv*) = A (*hv* − Eg)^1/2^ [55]. As presented in Figure 7c, the Eg value of PMo_10_V_2_O_40_ and MIL-101(Fe) was 1.98 eV and 1.90 eV, respectively. Moreover, the Mott–Schottky diagrams were analyzed to further obtain the conduction band energy (E_CB_) of pure PMo_10_V_2_O_40_ and MIL-101(Fe) (Figure 7d), which was 0.17 V and −0.08 V, respectively. Therefore, the valence band energy (E_VB_) of MIL-101(Fe) and PMo_10_V_2_O_40_ was 2.15 V and 1.82 V according to the formula of E_VB_ = E_CB_ + E_g_, respectively.

**Figure 8:**
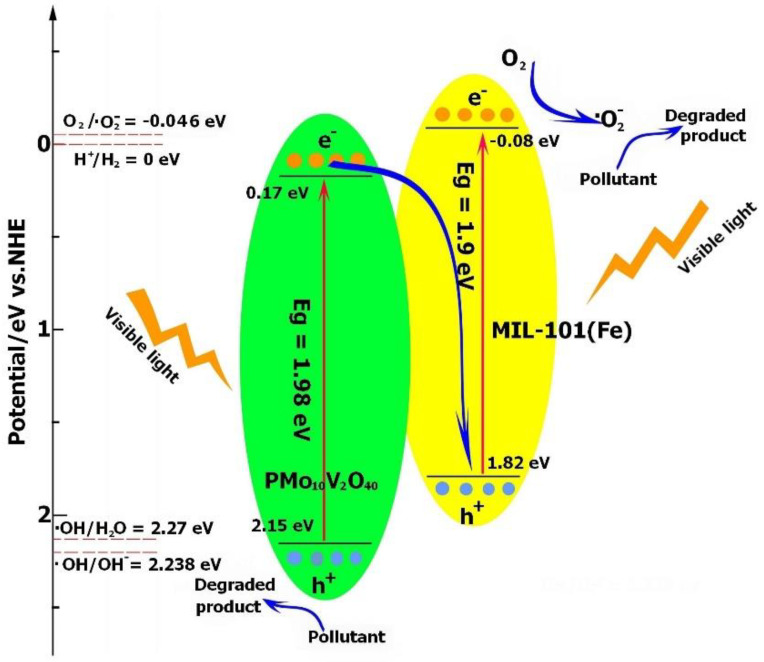
Proposed visible light photocatalytic mechanism of PMV_2_ for TC degradation.

Based on the analyses of the band structures of MIL-101(Fe) and PMo_10_V_2_O_40_, the photogenerated electrons and holes were transferred through the Z-scheme category. As demonstrated in Figure 8, the photoproduced electrons from the CB of PMo_10_V_2_O_40_ were transported to the VB of MIL-101(Fe) and combined with the holes, leading to more electrons and holes accumulating in the CB of MIL-101(Fe) and VB of PMo_10_V_2_O_40_, respectively. The electrons will accumulate in the CB of MIL-101(Fe) and reduce O_2_ to generate the ·O_2_^−^ radical (O_2_/·O_2_^−^ = −0.046 V vs. NHE). Because the VB of PMo_10_V_2_O_40_ exhibited a lower potential (2.15 V) than the reduction potential of ·OH/H_2_O (2.27 V) or ·OH/OH^−^ (2.38 V) [56], the holes in PMo_10_V_2_O_40_ were not sufficient to oxidize H_2_O or OH^−^ to generate ·OH, and they participated in the oxidation of pollutants directly. Therefore, *h*^+^ and ·O_2_^−^ were responsible for the degradation process.

## 4. Conclusions

In summary, a series of transition metal-substituted POMs were encapsulated into an MOF matrix by a simple one-pot reaction, resulting in the POM@MOF adsorbents. The introduction of POM into the MOF matrix can not only significantly enhance the adsorption capacity of the isolated MIL-101, but also endow the material with high performance for the photodegradation of TC. As a result, the adsorption capacity of PMV_2_ can reach as high as 912.5 mg·g^−1^, significantly superior to that of commonly used adsorption materials, such as activated carbon and graphene oxide. The complete photodegradation of adsorbed species induces the facile photoregeneration of these composites for multiple reusability. PMV_2_ represents the first POM@MOF composite for efficiently adsorbing and degrading antibiotics. This work highlights a new strategy for constructing highly efficient adsorbents by combining adsorption ability and photodegradation ability into one material.

## Data Availability

Not applicable.

## Supplementary Materials

The following supporting information can be downloaded at: https://www.mdpi.com/article/10.3390/molecules27113404/s1. 1. Experiments. 2. Spectral data. Figure S1: FT-IR spectra of PMo12O40, MIL-101 and PM. Figure S2: FT-IR spectra of of PMo11VO40, MIL-101 and PMV. Figure S3: FT-IR spectra of PMo10V2O40, MIL-101, and PMV2. Figure S4: Elemental mapping images of MIL-101(Fe). Figure S5: Elemental mapping images of PM. Figure S6: Elemental mapping images of PMV. Figure S7: The UV-Vis spectra of 20 mg·L^−1^ TC during the adsorption with 2 mg MIL-101 (a); PM (b); PMV (c); and PMV2 (d). Table S1: Adsorption capacity of TC with the reported and commercial adsorbents. Figure S8: The UV-Vis spectra of 40–10 mg·^−1^ TC during the photocatalytic degradation with PMV2. 3. Stability. Figure S9 UV-vis spectra of (a) PMo12O40, MIL-101 and the PM solution, (b) PMo11VO40, MIL-101 and PMV solution, (c) PMo10V2O40, MIL-101 and the PMV2 solution obtained by the centrifugation. Materials (1 mg) were immersed in 20 mL H2O. After 1 day, the materials were isolated by centrifugation. The resulting solutions were measured by UV-vis spectroscopy. Figure S10: XRD patterns of PMV2. Figure S11: Four cycles for degradation of TC with PMV2. Figure S12: The estimated band gaps of the prepared PM, PMV and PMV2 photocatalyst.
