# Supplementary material for: Filling Polyoxoanions into MIL-101(Fe) for Adsorption of Organic Pollutants with Facile and Complete Visible Light Photocatalytic Decomposition
Source: Molecules. 2022 May 25;27(11):3404. doi: 10.3390/molecules27113404 (PMC9182053; doi:10.3390/molecules27113404)
Supplement: Supplementary file 1 [file molecules-27-03404-s001.zip › molecules-1703348-supplementary.pdf]

Filling Polyoxoanions into MIL-101(Fe) for Adsorption of Organic Pollutants with Facile and Complete Visible Light Photocatalytic Decomposition

Qing Lan[a], Sujuan Jin[a], Bohan Yang[a], Zhi-Ming Zhang[b], Xuyang Li[a], Haiquan Xie[a,*], Xiaoli Jin[a], Huan Zhang[c,*], Qiang Zhao[a]

[a]*Engineering Technology Research Center of Henan Province for Solar Catalysis, College of Chemistry and Pharmaceutical Engineering, Nanyang Normal University, Nanyang 473061, China;*
[b]*Institute of New Energy Materials & Low Carbon Technology, School of Materials Science & Engineering, Tianjin University of Technology, Tianjin 300384, China;*
[c]*School of Science, Tianjin University of Science & Technology, Tianjin 300457, China.*

*Corresponding author:
E-mail address: Xie-hq@163.com (X. H. Q.), zhanghuan50423@163.com (H. Z.).

1. Experiments

1.1 Materials and methods

All the raw chemicals were obtained commercially and used without further purification. The $H_3PMo_{12}O_{40}$, $H_4PMo_{11}VO_{40}$, $H_5PMo_{10}V_2O_{40}$, and MIL-101 were prepared according to literature procedures, and identified by using IR spectroscopy. The powder X-ray diffraction (XRD) pattern was collected by using a Rigaku D/max-2550 diffractometer with $Cu_{k\alpha}$ radiation in the angular range 2θ 5°-50° at 293K. FTIR spectra were recorded on an Alpha Centaurt FT/IR spectrophotometer with KBr pellets in the range of 400-4000 cm^{-1} at room temperature. The thermogravimetric analysis (TGA) was performed on a Perkin-Elmer TGA7 instrument in flowing N_2 with a heating rate of 10 ℃ $\cdot min^{-1}$. Nitrogen adsorption-desorption isotherms was tested by using a Micrometrics ASAP-2020 Mautomatic specific surface area and porous physical adsorption analyzer at 77 K. UV-Vis absorption spectra were determined on a 756 CRT UV-Vis spectrophotometer.

1.2 Fabrication of MIL-101(Fe)

$FeCl_3\cdot 6H_2O$ (1.33 g, 4.90 mmol) was dissolved in DMF (50 mL), H_2bdc (0.41 g, 2.48 mmol) was added to this brown solution and stirred at room temperature for 10 min. The resulting mixture was divided into 10 portions and transferred into a 20 mL teflon lined autoclave and heated at 383K for 24 h. After cooling slowly to ambient temperature, the brown powder was centrifugal separation, and thorough washed with distilled water and DMF. The products were dried at 353 K under vacuum.

1.3 Synthesis of composite POM@MIL-101(Fe)

The composite POM@MIL-101 was synthesized by adding 0.2 g POM ($H_3PMo_{12}O_{40}$, $H_4PMo_{11}VO_{40}$ or $H_5PMo_{10}V_2O_{40}$) into the mixture of MOF precursors, which was stirred for 0.5 h. The following experimental procedure is identical with that of MIL-101(Fe).

2. Spectral data

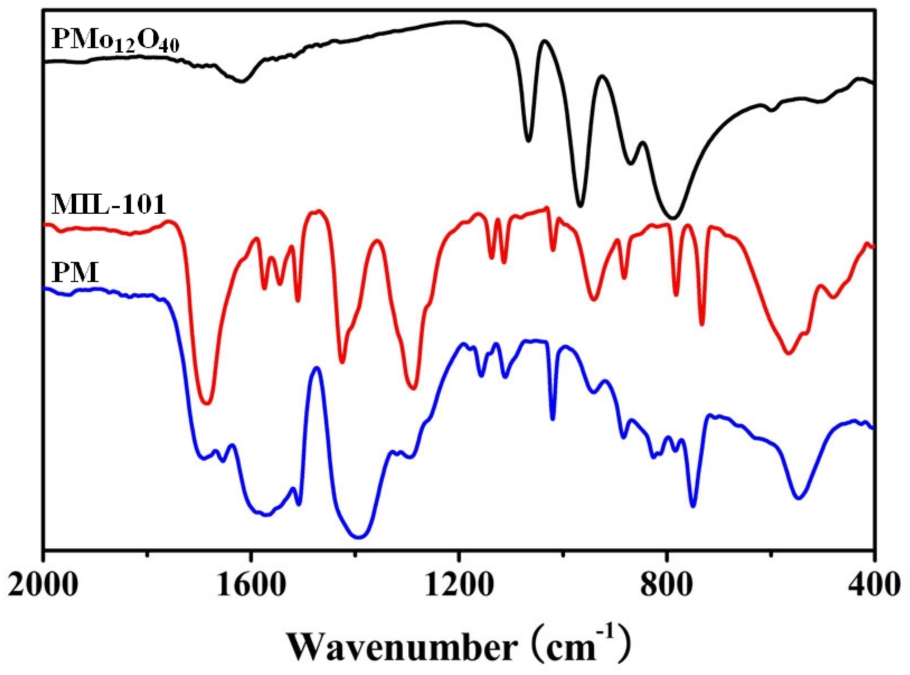

Figure S1 FT-IR spectra of $PMo_{12}O_{40}$, MIL-101 and PM.

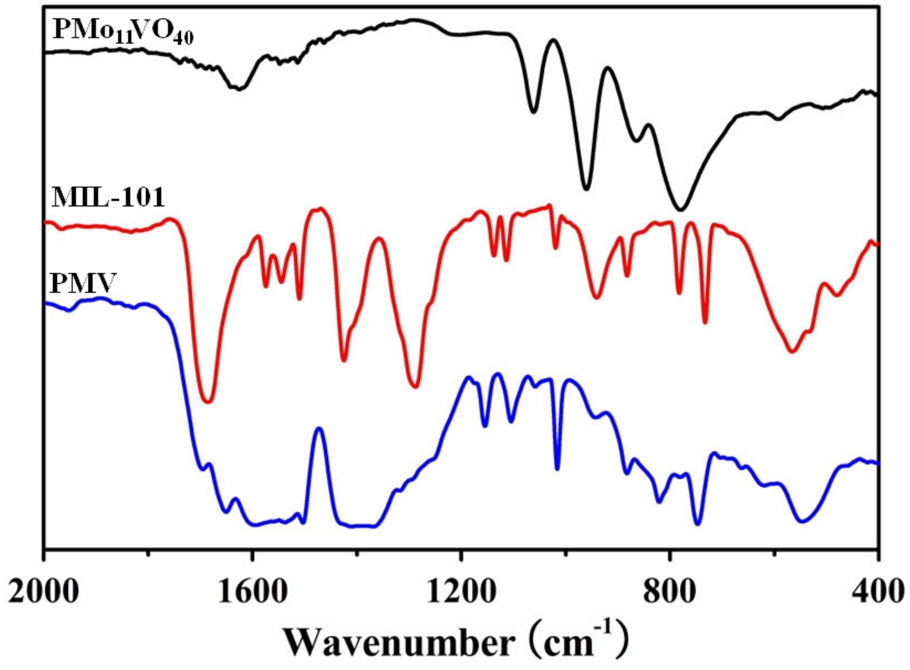

Figure S2 FT-IR spectra of of $PMo_{11}VO_{40}$, MIL-101 and PMV.

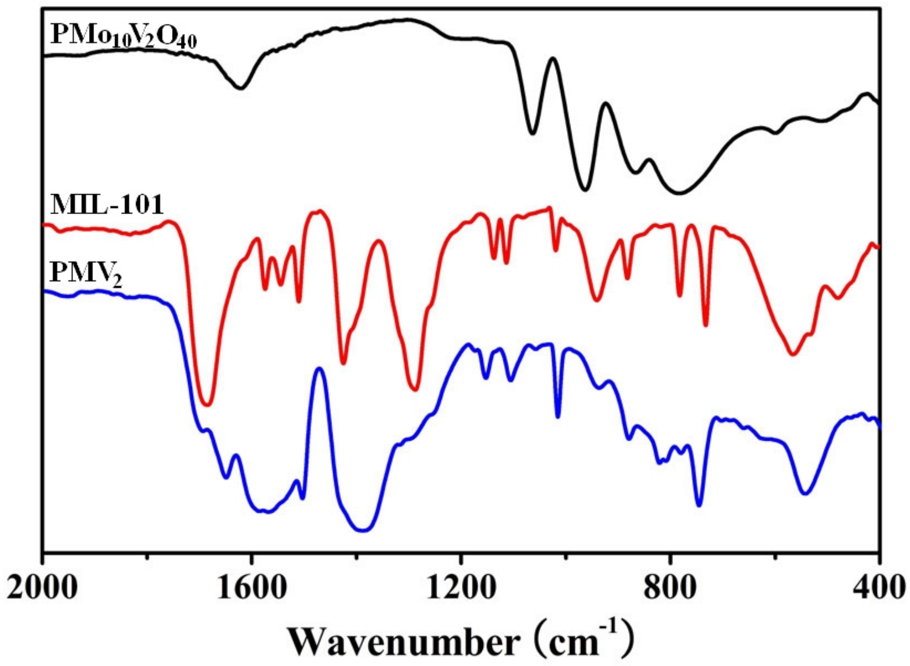

Figure S3 FT-IR spectra of $PMo_{10}V_2O_{40}$, MIL-101, and PMV_2.

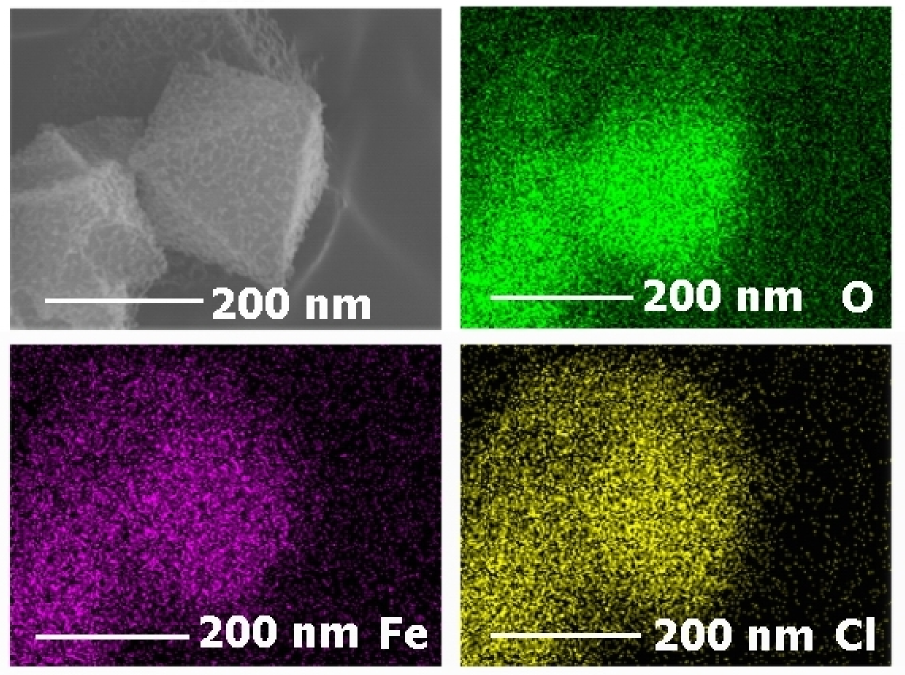

Figure S4 Elemental mapping images of MIL-101(Fe).

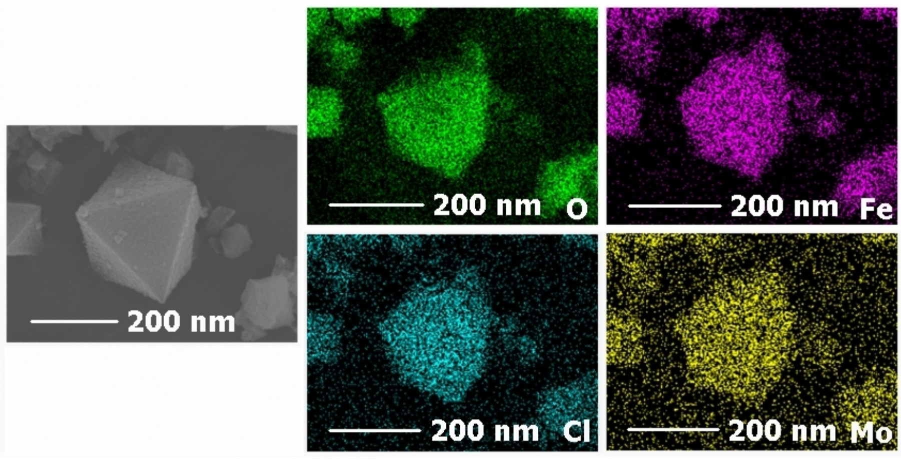

Figure S5 Elemental mapping images of PM.

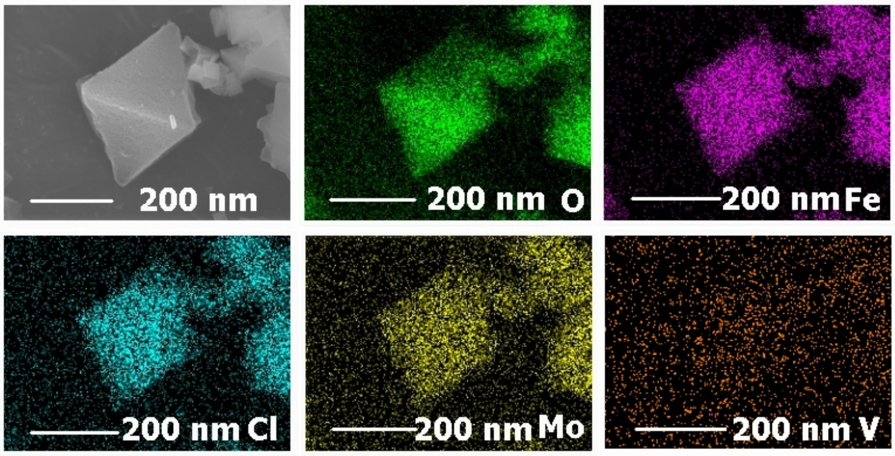

Figure S6 Elemental mapping images of PMV.

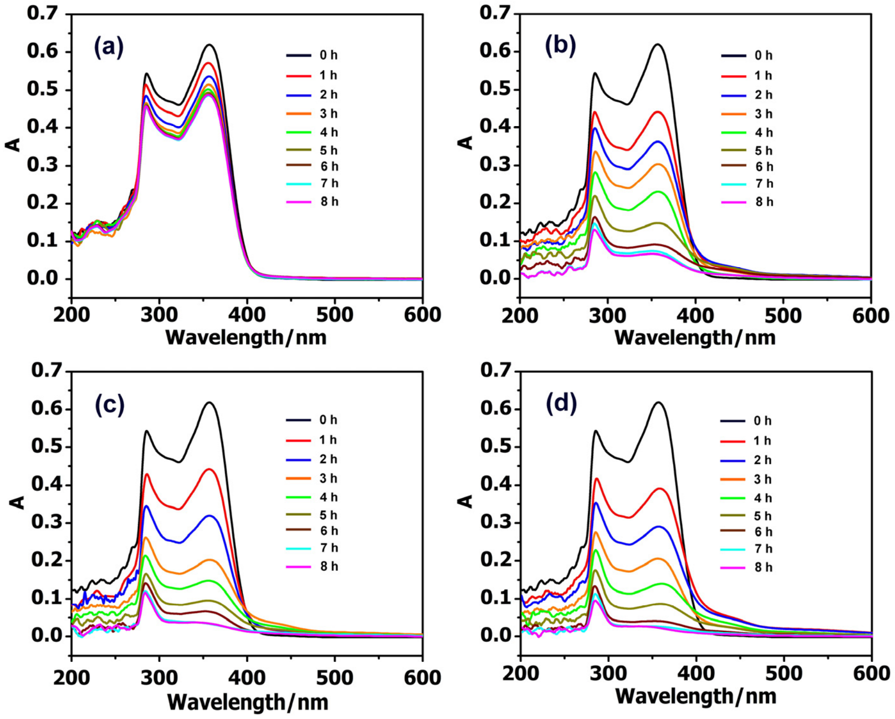

Figure S7 The UV-Vis spectra of 20 mg·L^{-1} TC during the adsorption with 2 mg MIL-101 (a); PM (b); PMV (c); and PMV$_2$ (d).

Table S1. Adsorption capacity of TC with the reported and commercial adsorbents.

Absorbent	q_{max} (mg·g^{-1})	Time (h)	References
Graphene oxide	313	2.5	18
Smectite	462	2	19
H_3PO_4-acdivated carbon	308	24	20
Fe modifed zeolite	200	6.5	21
UiO-66	23.1	6	22
C_3N_4-ZIF-8(ZC) composite	420	3	23
PM	647.6	8	This work
PMV	748.9	8	This work
PMV_2	839.5	8	This work

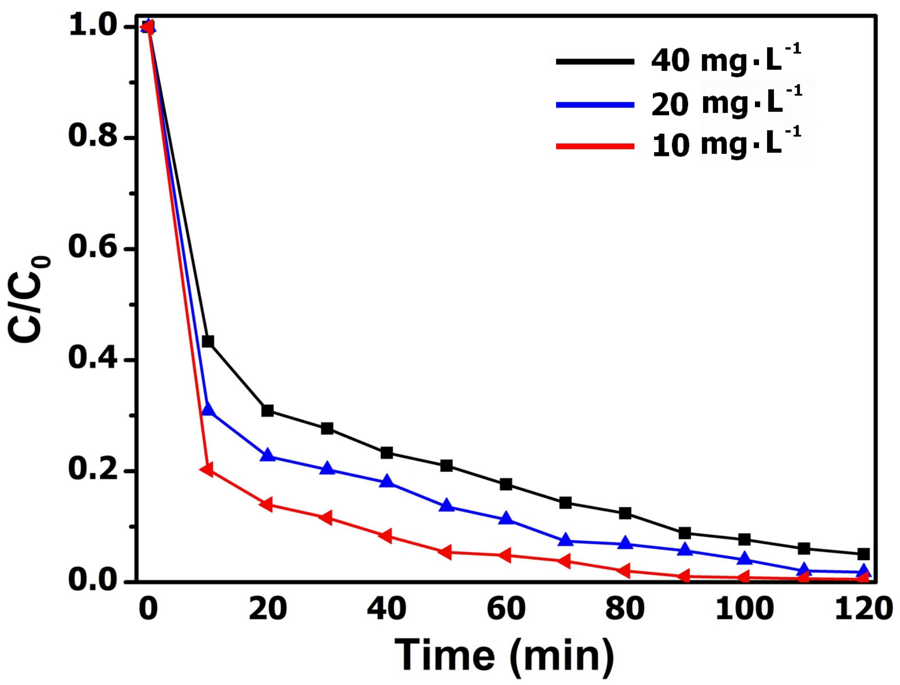

Figure S8 The UV-Vis spectra of 40-10 mg·L^{-1} TC during the photocatalytic degradation with PMV_2.

3. Stability

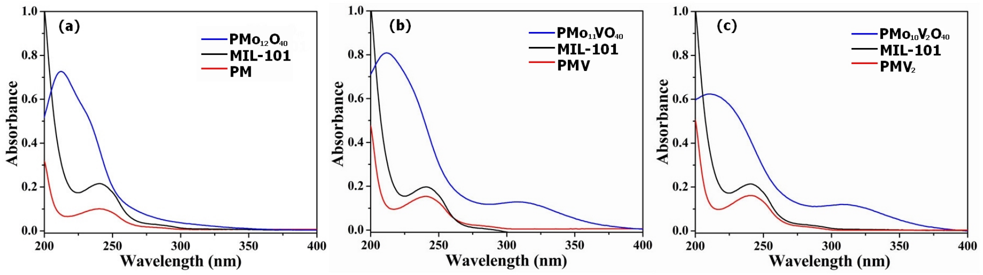

Figure S9 UV-vis spectra of (a) $PMo_{12}O_{40}$, MIL-101 and the PM solution, (b) $PMo_{11}VO_{40}$, MIL-101 and PMV solution, (c) $PMo_{10}V_2O_{40}$, MIL-101 and the PMV_2 solution obtained by the centrifugation. Materials (1 mg) were immersed in 20 mL H_2O. After 1 day, the materials were isolated by centrifugation. The resulting solutions were measured by UV-vis spectroscopy.

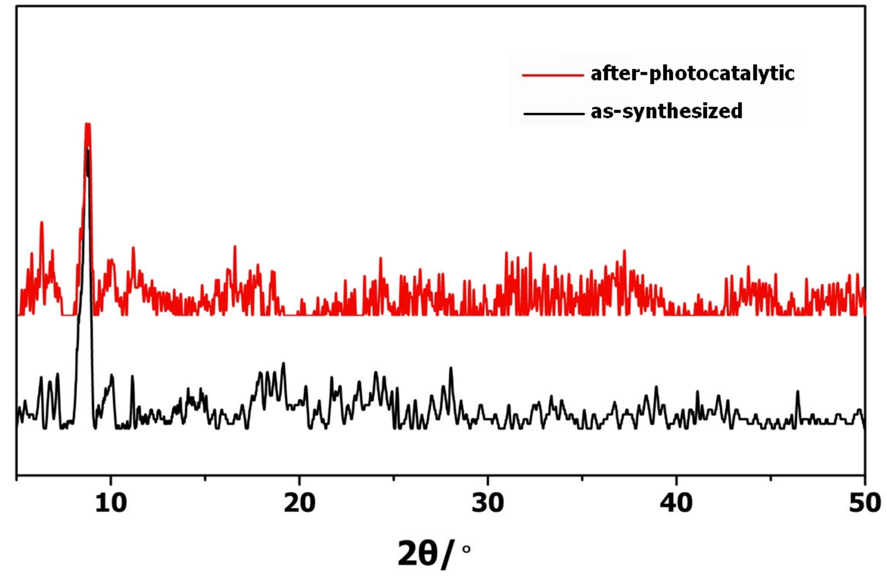

Figure S10 XRD patterns of PMV_2.

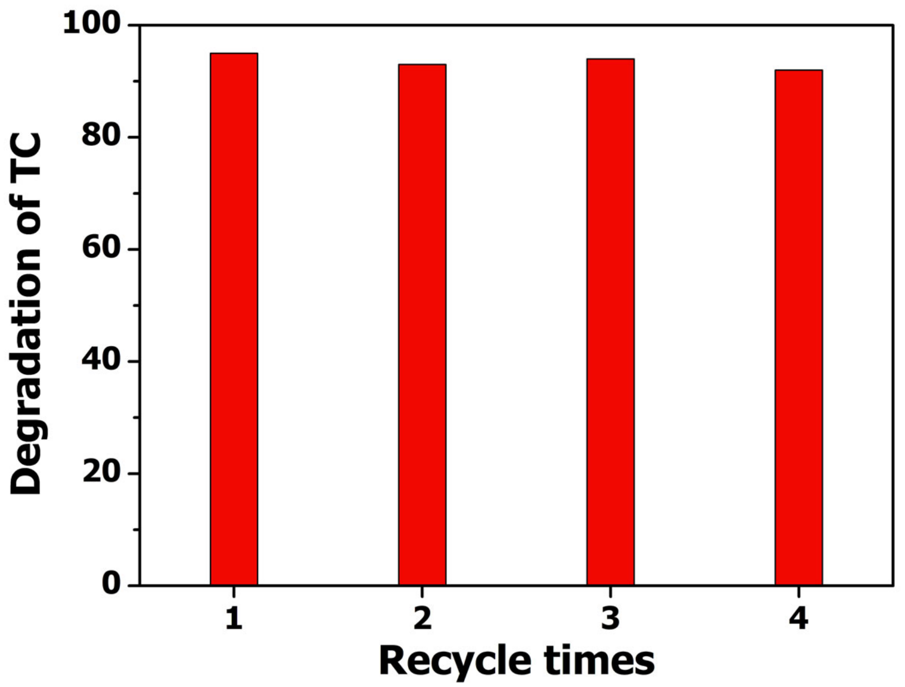

Figure S11 Four cycles for degradation of TC with PMV_2.

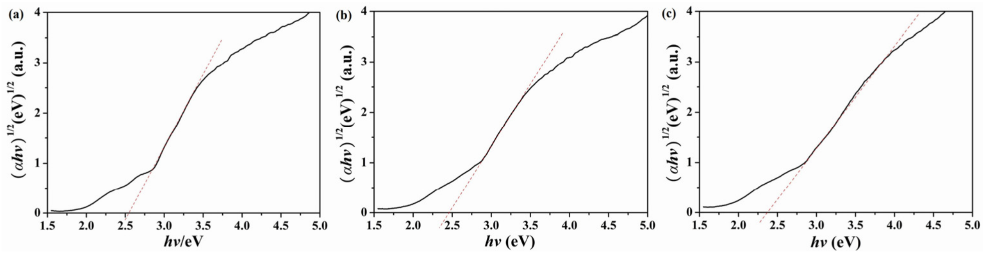

Figure S12 The estimated band gaps of the prepared PM, PMV and PMV_2 photocatalyst.
